# Supplementary material for: Anaerobic Digestion as an Alternative to Improve the Industrial Production of MnP Economically and Environmentally Using Olive Mill Solid Waste as the Substrate
Source: Foods. 2025 May 28;14(11):1918. doi: 10.3390/foods14111918 (PMC12155261; doi:10.3390/foods14111918)
Supplement: Supplementary file 1 [file foods-14-01918-s001.zip › foods-3602850-supplementary.pdf]

foods MDPI

Supplementary material

S.1 General considerations for the construction of the data inventory for each alternative.

The selected functional unit corresponds to the production of 1 kg of Manganese peroxidase. The functional unit in mass (kg) was selected according to a literature review of life cycle assessment of enzyme production.

The equivalence of the Manganese peroxidase liquid solution product to kg of Manganese peroxidase was calculated using a conversion factor from enzyme activity (U of MnP/L) to mass of MnP (Kg of MnP). This was based on the results obtained in the life cycle assessment of Manganese peroxidase production, using wheat straw as carbon source [22].

S.1.1 Production of MnP using the alternative 1.

Mixer (1). The synthetic culture medium is prepared in water with a modified kirk's medium according to the report by Acevedo et al. [55], which contains a concentration of 10 g/L glucose, 2 g/L peptone, 2 g/L KH_2PO_4, 0.5 g/L $MgSO_4$, 0.1 g/L $CaCl_2$, 2 mg/L thiamine and 0.0027 mg/L metal salts. The culture medium was adjusted to pH 4.5 [64]. The electrical requirement of the mixer was estimated according to agitator design criteria [65].

Sterilization (2). Sterilization was carried out at a temperature of 121°C for 15 minutes [64].

Fermentation (3). The sterilized culture medium was inoculated with *A. discolor* at a dose of 1.5 mg of dried fungus/L [2,16]. The fermentation was carried out for 15 days at a temperature of 30°C [55]. Biogenic CO_2 is produced during fermentation. Fermentation yields were assumed to be 0.45 kg glucose/m^3 d [66].

Centrifugation final culture (4). A Westfalia centrifuge with a nominal power of 15 kW was selected, with a processing capacity of 5 m^3/h. The efficiency of centrifugation was assumed to yield a residual biomass composed of 30% dry matter and 70% moisture. The enzymatic liquor obtained contains an enzymatic activity of 289 U of MnP/L [55], equivalent to 0.25 kg of MnP per m^3 of prepared synthetic culture medium, according to the conversion factors calculated in the research [22].

Ultrafiltration (5). The electrical efficiency for ultrafiltration was estimated according to the literature with a requirement of 1.5 kWh/m^3 [67]. The concentration factor used was 18-times to obtain concentrated MnP at an enzyme activity of 5,000 U MnP/L [68]. Thus, 92% of the liquid volume of input to the ultrafiltration corresponds to liquid effluent to treatment.

Thermal and chemical treatment (6). The residual biomass is thermally treated at 90°C [8] and a dosage of 20% CaO w/w is assumed for the chemical treatment. At this stage the soil improver for agricultural use is generated [8].

Boiler (7). The water, steam and diesel requirements in the boiler were estimated according to the heat required in the sterilization, fermentation and heat treatment stages. For sterilization, superheated steam at 130°C inlet and 98°C outlet was assumed. In fermentation and thermal treatment, water inlet at 98°C and outlet at 80°C. A boiler efficiency of 80% and a lower heating value of diesel of 43,100 KJ were assumed. Emissions to air were based on the emission factors reported for boilers by the Circular Economy and Water Service of Navarra, where the emission factors were 12 g CO/GJ Diesel, 74.1 kg CO_2/GJ Diesel, 0.6 g N_2O/GJ Diesel and 93 g SO_2/GJ Diesel.

S.1.2 Production of MnP using the alternative 2.

For this alternative the process units are the same as in alternative 1 (point S.1.1), where the only changes correspond to the substitution of a synthetic culture medium for OMSW.

Mixer (1). OMSW as substrate was used from the extra virgin Olive oil producing industry "Olivares de Quepú", Talca, Chile. The characterization of OMSW is shown in Table S1. The concentration of the culture medium was 69.3 g of OMSW/L without pH adjustment [2]. Electrical requirements were the same as the alternative 1.

Table S1. Olive mill solid waste characterization

Total solids (%w/w)	38.5
Volatile solids (%w/w)	34.5
Total organic carbon (%w/w)	29.2
Chemical oxygen demand (mg/L)	87,867
Total nitrogen (mg/Kg)	282.9
Carbon (%w/w)	48.4
Nitrogen (%w/w)	0.84
pH	5.1
C/N ratio	58

Sterilization (2). This was carried out at 121°C for 15 minutes [2].

Fermentation (3). The dose of *A. discolor* was assumed to be the same as the alternative 1. The fermentation was carried out for 15 days at a temperature of 25°C [2]. Biogenic CO_2 is produced during fermentation.

Centrifugation final culture (4). Centrifugation efficiency and electricity required was the same as in alternative 1. The enzymatic liquor obtained contains an enzymatic activity of 119 U of MnP/L [2], equivalent to 0.1 kg of MnP per m^3 of prepared Olive mill solid waste, according to the conversion factors calculated in the research [22].

Ultrafiltration (5). The electrical efficiency used was the same as the alternative 1. 43 times concentration of the enzyme liquor is required to obtain a concentration of 5,000 U of MnP/L. Thus, 82% of the liquid volume of input to the ultrafiltration corresponds to liquid effluent to treatment.

Thermal and chemical treatment (6). The temperature and CaO dosage to obtain a soil improver was the same as the alternative 1.

Boiler (7). The boundary conditions for water, diesel and boiler air emissions requirements were the same as the alternative 1.

S.1.3 Production of MnP using the alternative 3.

This alternative incorporates anaerobic digestion to supply the energy requirements. It uses the same MnP production method as the alternative 2.

Mixer (8). The residual biomass concentration is adjusted to 9% w/w with liquid effluent to treatment [69]. The electrical requirement was the same as the previous alternatives.

Anaerobic digestion (9). The biogas production yield was determined in the experimental assays in point 3.1, achieving 171 mL CH_4/kg volatile solids. The electrical requirement was assumed to be similar to the mixers of the previous alternatives. The 200 kJ of thermal energy per kg of OMSW fed to the AD was consumed to keep the operating temperature of the reactor [69].

Cogeneration (10). A lower heating power of methane equal to 36,000 KJ/m^3 was assumed [57]. The cogeneration conversion efficiency was 33% for electricity and 55% for heat [69].

Centrifugation digestate (11). The efficiency of centrifugation it was assumed that a dehydrated digestate with 30% dry matter and 70% moisture is obtained. 56% of the liquid volume entering the centrifuge corresponds to liquid effluent to treatment. The electrical efficiency of digestate centrifugation was assumed to be 3.5 kWh/Ton of digestate [69].

Composting (12). During composting, the moisture is reduced to 35% and gases such as CH_4, N_2O and NH_3 are emitted to the air [69]. Emission factors were 1.83, 0.075 and 0.406 kg per ton of raw material, respectively [70].

S.2 Assumptions and limitations

- Chemical inputs that represented less than 1% in alternative 1 were not considered in LCA because they were insignificant (Thiamine, Metallic salts and HCl).
- Chemical inputs such as: Peptone, CaO and KH_2PO_4 are not modeled in the Ecoinvent database. Peptone and CaO represent 12% and 15% of the total chemicals used in SCM, respectively. While, KH_2PO_4 represents 12% of the chemical inputs. However, for KH_2PO_4 it was possible to elaborate a life cycle inventory of the stoichiometric production of 1 kg of KH_2PO_4 (Table S3).
- It is assumed that in fermentation 12.8% of the carbon is transformed into biogenic CO_2 [71].
- The seed fermentation phase was not included in the analysis, as it only represents between 1% and 3% of the overall impacts [22,72,73].
- Construction and decommissioning of treatment plants are excluded in all cases due to their long useful life, therefore, impacts can be considered negligible with respect to the functional unit [69].

S.3 System expansion approach

Table S2 shows the credits associated with the products avoided for each production alternative of 1 kg of Manganese peroxidase.

For the soil improver and compost, equivalence ratios were calculated to avoid the production of 1 kg of ammonium nitrate fertilizer, one of the most widely used fertilizers in Chile, which contains 34% plant-available nitrogen [53]. It was assumed that 50% of the final nitrogen from the process would be available to the plants [74]. For the avoided electricity product, injection into the Chilean national grid was assumed. Heat was not considered as an avoided product, since there is no infrastructure for its use in Chile. Thus, it is assumed that it dissipates in nature without significant impact.

Table S2. Credits associated with avoided products for each alternative.

MnP production	Outcomes	Credits for avoided products	Equivalence ratio
Alternative 1	Soil improver	Ammonium nitrate[1]	33:1 (Kg)
Alternative 2	Soil improver	Ammonium nitrate[1]	81:1 (Kg)
Alternative 3	Electricity	Medium voltage-Chilean mix	1:1 (Kwh)
	Compost	Ammonium nitrate[1]	89:1 (Kg)

[1] Ammonium nitrate: 34% of nitrogen according to [53]; MnP: Manganese peroxidase liquid solution.

S.4 Life cycle inventory for potassium monophosphate

Table S3. Life Cycle Inventory (LCI) for the production of 1 kg of potassium monophosphate.

Reactants				Products		
Phosphoric acid	+	Potassium hydroxide	→	Potassium monophosphate	+	Water
**molecular weight**						
98.00 g/mol		56.11 g/mol		136.08 g/mol		18.02 g/mol
**stoichiometric formula**						
1 H_3PO_4	+	1 KOH	→	1 KH_2PO_4	+	1 H_2O
**mass balance (LCI)**						
0.72 kg		0.41 kg	→	1 kg	+	0.13 kg

S.5 Inventory data

Table S4. shows the global inventory for the three alternative productions considering 1 kg of MnP produced as a functional unit.

Table S4. Inventory data referred to 1 kilogram of Manganese peroxidase produced. n.a; not applied.

Item	Unit	Alternative 1	Alternative 2	Alternative 3
Input				
Water	kg	4,420	10,071	8,993
Synthetic culture medium*				
Glucose	kg	40	n.a	n.a
Peptone	kg	8	n.a	n.a
KH_2PO_4	kg	8	n.a	n.a
$MgSO_4$	kg	2	n.a	n.a
$CaCl_2$	kg	0.4	n.a	n.a
Thiamine	g	8	n.a	n.a
Mineral salts	g	0.01	n.a	n.a
HCl	g	29.2	n.a	n.a
Olive mill solid waste substrate*	kg	n.a	693	693
Anthracophyllum discolor	g	6	15	15
Electricity	kWh	33	80.7	0
CaO	kg	11.2	133	n.a
Diesel	kg	75	233	75
Output				
CO_2 biogenic	kg	2.04	25.8	25.8

Liquid effluent to treatment	m^3	3.68	8.2	8.6
Manganese peroxidase liquid solution	L	215	196	196
Soil improver*	kg	67.6	800	n.a
Compost*	kg	n.a	n.a	483
Electricity	kWh	n.a	n.a	158[1]
Heat	MJ	n.a	n.a	0
Emission to air composting				
CH_4	kg	n.a	n.a	0.900
NH_3	kg	n.a	n.a	0.200
N_2O	kg	n.a	n.a	0.036
Emission to air boiler				
CO	g	38.7	120.5	38.8
CO_2	kg	239	744	240
N_2O	g	1.9	6.0	1.9
SO_2	g	300	934	301

[1] Net electricity production after subtracting total electricity consumed from electricity produced.

(*) Results expressed on a dry basis
